# Preoperative Heart Rate Variability as Predictors of Vagus Nerve Stimulation Outcome in Patients with Drug-resistant Epilepsy

**DOI:** 10.1038/s41598-018-21669-3

**Published:** 2018-03-01

**Authors:** Hong-Yun Liu, Zhao Yang, Fan-Gang Meng, Yu-Guang Guan, Yan-Shan Ma, Shu-Li Liang, Jiu-Luan Lin, Long-Sheng Pan, Ming-Ming Zhao, Wei Qu, Hong-Wei Hao, Guo-Ming Luan, Jian-Guo Zhang, Lu-Ming Li

**Affiliations:** 1 National Engineering Laboratory for Neuromodulation, School of Aerospace Engineering, Tsinghua University, 100084 Beijing, China; 2 Department of Biomedical Engineering, Chinese PLA General Hospital, Fuxing Road, 100853 Beijing, China; 3 Beijing Neurosurgical Institute, 100050 Beijing, China; 4 Neurosurgery, Beijing Tian Tan Hospital Capital Medical University, 100050 Beijing, China; 5 Neurosurgery, Sanbo Brain Hospital Capital Medical University, 100093 Beijing, China; 6 Neurosurgery, Peking University First Hospital FengTai Hospital, 100071 Beijing, China; 7 Neurosurgery, Chinese PLA General Hospital, Fuxing Road, 100853 Beijing, China; 8 Neurosurgery, TsingHua University YuQuan Hospital, 100040 Beijing, China; 9 Neurosurgery, Navy General Hospital, 100048 Beijing, China; 10 Man-Machine-Environment Engineering Institute, School of Aerospace Engineering, Tsinghua University, 100084 Beijing, China; 11 Precision Medicine & Healthcare Research Center, Tsinghua-Berkeley Shenzhen Institute, 518055 Shenzhen, China; 12 Center of Epilepsy, Beijing Institute for Brain Disorders, 100069 Beijing, China

## Abstract

Vagus nerve stimulation (VNS) is an adjunctive treatment for drug-resistant epilepsy (DRE). However, it is still difficult to predict which patients will respond to VNS treatment and to what extent. We aim to explore the relationship between preoperative heart rate variability (HRV) and VNS outcome. 50 healthy control subjects and 63 DRE patients who had received VNS implants and had at least one year of follow up were included. The preoperative HRV were analyzed by traditional linear methods and heart rhythm complexity analyses with multiscale entropy (MSE). DRE patients had significantly lower complexity indices (CI) as well as traditional linear HRV measurements than healthy controls. We also found that non-responders_0_ had significantly lower preoperative CI including Area 1–5, Area 6–15 and Area 6–20 than those in the responders_0_ while those of the non-responders_50_ had significantly lower RMSSD, pNN50, VLF, LF, HF, TP and LF/HF than the responders_50_. In receiver operating characteristic (ROC) curve analysis, Area 6–20 and RMSSD had the greatest discriminatory power for the responders_0_ and non-responders_0_, responders_50_ and non-responders_50_, respectively. Our results suggest that preoperative assessment of HRV by linear and MSE analysis can help in predicting VNS outcomes in patients with DRE.

## Introduction

Epilepsy, characterized by recurrent and unprovoked seizures, is one of the most common and serious, chronic neurological disorders that affects around 65 million people worldwide^1^. Appoximately 30–40% of these patients who fail to attain seizure control with adequate trials of two tolerated, appropriately chosen and used antiepileptic drug (AED) schedules are considered as drug-resistant epilepsy (DRE)^2^. Even after complete preoperatively evaluations, only 10–50% of these DRE patients are suitable for conventional craniotomy surgery^3^. Vagus nerve stimulation (VNS) has been widely accepted as an adjunctive therapy for DRE with more than 115,000 devices implanted in about 80,000 patients worldwide^4,5^.

Despite the growing application of VNS, the efficacy varies substantially due to clinical factors including epilepsy type, etiology, AED regimens, severity of the epilepsy and usually does not result in complete cessation of seizures^6^. Furthermore, it is still difficult to predict which patients respond to what extent to VNS treatment. Prognostic biomarkers will be very useful for counselling patients and predicting the VNS seizure control outcome. Biomarkers indicating a good prognosis will help potential responders to avoid treatment before they receive an effective VNS system while a low likelihood to response could indicate that other therapy options may be more useful and will avoid the high cost of VNS treatment and protect them from possible risks induced by implantation and chronic stimulation. The identification of predictors could also help promote understanding of the neurobiological mechanisms of the effect of VNS on seizure control.

Studies have shown that epilepsy and recurrent seizures can lead to interictal and ictal dysfunction of cardiac autonomic regulation, represented as heart rate variability (HRV) alterations^7–9^. However, conventional linear algorithms are often applied to calculate measures of HRV, even though the modulation of the autonomic nervous system (ANS) on cardiac activity is considered to be a nonlinear physiological process with non-stationary property, and their prognostic values for VNS outcome in patients with DRE were unclear^10–12^. Since applying linear approaches to HRV signals may introduce intrinsic computational errors^13^, efficient methods for characterizing the complex non-linear dynamics of the heart rate remain to be established. Recently, Multiscale entropy (MSE) analysis has been introduced to quantify the complexity of physiological data sets over different temporal scales, offering more differentiated and exact insights into the control mechanisms underlying nonlinear dynamics^10,11^. At present, MSE has been extensively used to analyze several biological signals for diagnosis, classification, risk stratification, and prognosis of diseases such as stroke, heart failure, primary aldosteronism, patients undergoing peritoneal dialysis, Alzheimer’s disease, autism spectrum disorder and Parkinson’s disease^14–21^. However, MSE analysis of heart rate dynamics in DRE patients and its association with VNS outcome have not been studied previously. The present study aimed to investigate whether preoperative HRV, as quantified by traditional linear measurements and non-linear heart rhythm complexity, are predictors for seizure reduction of VNS treatment in patients with DRE.

## Methods

### Study design and participants

The patients had undergone VNS surgery at seven hospitals (Beijing Tiantan Hospital Capital Medical University, Sanbo Brain Hospital Capital Medical University, TsingHua University YuQuan Hospital, Peking University First Hospital FengTai Hospital, Chinese PLA General Hospital, First Affiliated Hospital of PLA General Hospital and Navy General Hospital) between August 13, 2014 and December 31, 2015 and were undergoing their one year follow up evaluation. All patients underwent complete presurgical evaluations including long term (interictal and ictal) video-EEG, 24-hour electrocardiography (ECG) recordings, MRI or PET, and comprehensive clinical as well as neuropsychological assessments as part of their diagnoses to ascertain that their DRE was not suitable for traditional epileptic craniotomy surgery^1,2^.

The inclusion criteria were: (1) 5–60 years old, (2) having tried at least two appropriate AED tested to tolerance or to blood levels at the upper end of the target range of which at least 2 had been tolerated at the normal dose, (3) at least 1 seizure per month, (4) in good health except for the epilepsy, (5) with a minimum mental state examination (MMSE) score ≥18 (no severe cognitive impairment). The exclusion criteria were: (1) the MRI or PET results indicating that the epilepsy was caused by intracranial space-occupying lesions, (2) tumors, cardiopulmonary anomalies, progressive neurological diseases, asthma, mental disease, or any other known disease that may have affected the ANS function, (3) alcohol addiction, smoking, and sleep-related breathing disorders and (4) a history of medication that may have impacted the autonomic function. Healthy control subjects were selected according to the age range and gender ratio of the included DRE patients. All the healthy had no medication or other disease affecting the ANS function based on their medical history and physical examination results.

Participants with a history of any known disease, sleep disorders and/or medication that affected the ANS were excluded from the study to avoid the potential influence on the HRV. The observed variables included their demographic data, seizure type, epilepsy duration, etiology, age at VNS surgery, seizure frequency, number of AED used, total dose of AED per day, presurgical MRI or PET findings, ictal scalp video-EEG characteristic and preoperative ECG recordings. Three months prior to the VNS surgery and during the one year follow up period after the VNS treatment, the number and doses of the AED regimens were kept unchanged. The patients or their family members were asked to keep diaries to document time, duration and type of each daily physical activity and possible seizures during the recording period. Baseline and one year follow up seizure frequency were also determined based on their diaries. This study was approved by the Institutional Review Committee of Beijing Tiantan Hospital Capital Medical University, and all subjects, or parents/guardians of the subjects, gave informed consent in written form including for the collection of their information and usage for research. The methods in the study were carried out in accordance with the approved guidelines.

### Vagus nerve stimulation

The VNS system (PINS Inc., Beijing, China) was implanted to stimulate the left vagus nerve of the DRE patients. The details of the surgical procedure have been described elsewhere^4,5^. The VNS generator was turned on about 2 weeks after implantation with initial settings being a current amplitude of 0.2 mA, frequency of 30 Hz, pulse width of 500 μs, signal on time of 30 s, and signal off time 5 min. Adjustments were made at intervals of about 2 weeks until the stimulation reached 1.0 mA. This was followed by 1 month intervals for the first 4 months and then preceded by 4 month intervals. At each follow up visit, the output current was progressively increased by 0.2–0.3 mA until (1) the seizures were reduced by more than 50%, (2) the patient no longer tolerated the treatment, or (3) the current reached a maximum of 3.5 mA^5^.

The seizure frequency and side effects of the VNS treatment were evaluated at the 4 months, 8 months and one-year post-implantation during clinical visits. The assessed seizure reduction during the follow up period was used to determine the response to VNS therapy. The mean seizure frequency per month was calculated and the responders_0_ were defined as having any reduction in seizure frequency while responders_50_ had at least 50% seizure reduction.

### Ambulatory ECG recording and preprocessing

A 12-lead ambulatory ECG monitoring system (MIC-12H-3S, JincoMed, Beijing) with a digital sampling rate of 500 samples/second per channel was used to record consecutive 24-hour ECG for all the participants. The conventional ambulatory ECG configurations of leads V5, which provided a stable and reliable signal was selected as the principal analysis lead. Participants underwent 24-hour ECG monitoring in free-moving conditions and were asked to keep activity diaries to document time, duration and type of each daily physical activity and possible seizures during the recording period. All 24-hour Holter recordings were performed automatically by a PC-based acquisition system (SkyHolter, JincoMed, Beijing). The annotated files were then carefully inspected and corrected by technicians for extracting the RR intervals from leads II and V5. The ectopic beats were interpolated by its adjacent RR intervals for adjustment and correction. At least 50% of each 24-hour ECG recording had to be suitable for traditional HRV analysis for a record to be included in accordance with issued guidance^22^. Four-hour periods of RR intervals without exercise and naps within daytime (between 9AM and 5PM, 2 hours after the administration of AEDs) were selected from each recording for MSE analysis^10,11,16–18^. All ECG segments with a four-hour length were selected from the same period to reduce confounding effects of the circadian rhythm and physical activity^18^. Only subjects with recordings of more than 80% of qualified normal sinus beats were included for further analysis^16^.

### Time and frequency domain analysis

The HRV time domain measures included the mean RR intervals (Mean RR), standard deviation of the RR intervals (SDNN), square root of the mean of sum of squares of the differences between adjacent RR intervals (RMSSD), and pNN50. The latter the proportion of the NN50 (successive RR intervals differing by more than 50 ms) divided by the total number of RR intervals in the ECG recordings. The SDNN provides information about all the components contributing to the HRV during the recording period and is, therefore, a fairly global measure of HRV. The RMSSD and pNN50 reflects the cardiac parasympathetic control of the heart rate^22–24^. Fast Fourier transform was used to calculate the four main spectral components for the total power (TP) for the frequency range 0.0033–0.40 Hz; the very low frequency power (VLF) for the frequency range 0.0033–0.04 Hz; the low frequency power (LF) for the frequency range 0.04–0.15 Hz and the high frequency power (HF) for the frequency range 0.15–0.40 Hz. The VLF component is a major determinant of the physical activity and possibly reflects sympathetic activity, though its origin remains controversial^22^. The LF component reflects both sympathetic and parasympathetic control of the heart rate, while the HF component is generally interpreted as a marker of vagal activity and is respiration mediated^22^. The ratio of LF to HF (LF/HF) reflects the global sympatho-vagal balance or reflects the sympathetic activity^22^.

### MSE analysis

**Figure 1:**
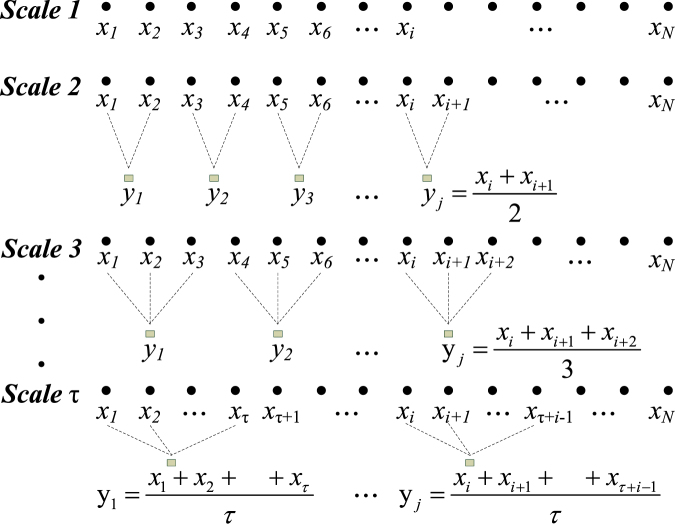
The illustration of the coarse graining procedure for scales 1 to τ.

The MSE method was used to analyze complexities of nonlinear and nonstationary signals in finite length time series. The method comprised of two procedures: (1) Coarse-graining the signals into different time scales. E.g. for a given time series $\{{x}_{i},L,{x}_{i},{\rm{L}}\,{x}_{N}\}$, multiple coarse-grained time series ${{\rm{y}}}_{j}^{(\tau )}$ were constructed by averaging the data points within non-overlapping windows of increasing scale factor, τ (Fig. 1). Each element of the coarse-grained time series was calculated according to the equation (1):

1 $${y}_{j}^{(\tau )}=\frac{1}{\tau }\sum _{i=(j-1)\tau +1}^{j\tau }{x}_{i},1\le j\le N/\tau $$

For scale 1, the time series y^(1)^ was simply the original time series. The length of each coarse-grained time series was taken as *N/τ*. (2) Sample entropy for each coarse-grained time series was quantified with values m = 2 and r = 0.15*SDNN, where r was the size of the cell utilized to coarse-grain the phase space, SDNN was the standard deviation of the four-hour period of RR interval time series, m was the embedding dimension, which was then plotted as a function of the scale factor τ. In depth details of this methodology have been previously described^10,11,16,17^.

**Figure 2:**
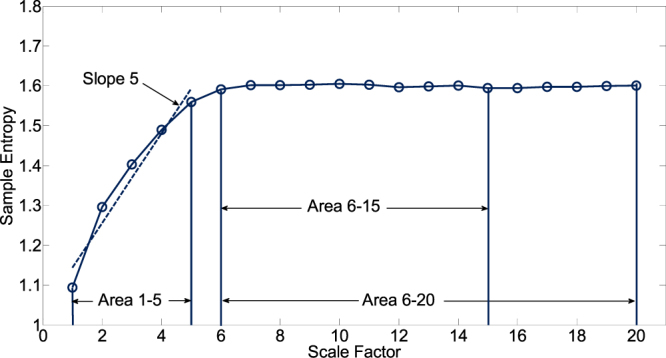
CI derived from MSE profile. The linear-fitted slope (Slope 5) and the area under MSE profile between scale 1 and 5 (Area 1–5) represent the complexity behavior in short scales. Long time scale complexities were quantified by the area under MSE profile between scale 6 and 15 (Area 6–15), and between scale 6 to 20 (Area 6–20).

The different features revealed from small and large scales in different groups of subjects has been used to assist the clinical categorization^11^. In order to present the complexity of ECG signal in a straight-forward manner and also to present the structural richness of information over multiple spatial and temporal scales, the complexity indices (CI) were quantified by curve fitting and calculating the area between the curve of MSE and the axis of scale factors^16,17^. The linear-fitted slope (Slope 5) and the area under MSE profile between scale 1 and 5 (Area 1–5) were calculated to quantify the complexity and to characterize the modulation pattern in short scales. Long time scale complexities were quantified by the area under the MSE profile between scale 6 and 15 (Area 6–15), and between scale 6 to 20 (Area 6–20), respectively (Fig. 2).

### Statistical analysis

Data are presented as mean ± standard deviation (SD) for continuous variables. Gaussian distribution and homogeneity of variance tests were applied to determine the distribution and homoscedasticity of sample data. As a result of the non-normal distribution and heterogeneity of variance of some sample data, a Mann-Whitney U test was applied to compare the responders_0_ and non-responder_0_. For single predictive variable analysis using qualitative or categorical variables, Fisher’s exact tests were applied for comparison between the two groups. The receiver operating characteristics (ROC) curve was created based on the sensitivity and specificity of the continuous variables in predicting seizure reduction of VNS treatment in patients with DRE. The area under the ROC curve (AUC) gave an estimate of the overall discriminate ability (AUC = 0.5 indicates no discrimination and an AUC = 1.0 indicates a perfect diagnostic test). The relationship between the seizure reduction (%) and the HRV measurements (Area 1–5, Area 6–15, Area 6–20, RMSSD, pNN50, VLF, LF, HF, TP and LF/HF) as well as the output current amplitude were tested by Pearson’s correlation analysis. Significant HRV indices in the Pearson’s correlation analysis were then analyzed by multivariable logistic regression test with stepwise subset selection to determine independent factors for predicting seizure reduction of VNS treatment. All statistical analyses were performed using SPSS version 20 software package (SPSS, Chicago, Ill, USA). All the p values were adjusted using the false discovery rate (FDR) method and a value of p < 0.05 was considered to indicate statistical significance.

## Results

### Study population

Eventually, 63 patients with DRE receiving VNS treatment and undergoing their one year follow up were enrolled. Eleven patients with DRE reported having experienced possible seizures during the preoperative 24-hour ECG recording, with ten patients having focal seizures with each seizure attack not exceeding 60 seconds. The ECG episodes with seizures were discarded to remove their effects on HRV measurements. However, the HRV for these eleven patients were not particularly different from those for the other patients; hence there is no reason to believe that the seizures significantly affected the results. Demographic data, clinical factors and physical activity of DRE patients and healthy control subjects are presented in Table 1 and Table S-1.

**Table 1:** Demographic data, clinical factors and physical activity types and duration of all study population. N. A = Not Available.

Variables	DRE patients (n = 63)	Healthy control subjects (n = 50)	*P* value
**Demographic data & clinical factors**			
Male/female	42/21	34/16	1.000
Age (year)	18.3 ± 8.8	21.0 ± 7.8	0.090
BMI(kg/m^2^)	22.0 ± 4.4	21.7 ± 3.3	0.617
Epilepsy duration (year)	11.1 ± 7.2	N.A	N.A
Seizures per month	95.1 ± 167.2	N.A	N.A
Number of AEDs	2.6 ± 1.2	N.A	N.A
Dose of AEDs per day (mg)	1677 ± 1007	N.A	N.A
**Daily activity**			
Sleeping overnight (hour)	8.4 ± 1.2	8.3 ± 1.1	0.209
Sitting quietly or lying quietly (hour)	3.5 ± 2.0	3.2 ± 1.0	0.899
Sitting busy (hour)	4.9 ± 1.8	5.1 ± 1.3	0.191
Light on-foot activities (hour)	5.1 ± 1.9	5.3 ± 1.3	0.209
Moderate on-foot activities (hour)	1.7 ± 1.0	2.1 ± 1.2	0.209
Strenuous activities (hour)	0.0 ± 0.0	0.0 ± 0.0	1.000
Very strenuous activities (hour)	0.0 ± 0.0	0.0 ± 0.0	1.000

The 63 patients included 42 men and 21 women ranging in age from 5 to 38 years at the time of VNS implantation. There were 55 (87.30%) patients (the responders_0_ group) that responded to VNS, with 34 (53.97%) patients having seizure reductions of at least 50% (the responders_50_ group) at the end of the one-year follow-up period. A total of 9 (14.29%) of the 63 patients became seizure-free after the one-year VNS treatment. In addition, no serious adverse events found in the patients with DRE. The characteristics and the amount of time the resonders_0_ and resonders_50_ spent on different types of daily physical activity exhibited no significant statistical differences (all p > 0.05) in comparison with those of the non-responders_0_ and non-responders_50_, respectively. The clinical variables including gender, age at VNS implantation, body mass index (BMI), epilepsy duration, seizure frequency, seizure type, etiology, number, daily dose and carbamazepine (CBZ)/oxcarbazepine (OXCBZ) regimens of the AED used showed no significant differences between the responders_0_ and non-responders_0_ as well as the responders_50_ and non- responders_50_ (Table 2). The stimulation parameters during the VNS ON condition were patient-specific at the end of one year follow up period (Table S-1). These values were the habitual therapeutic parameters of each patient that gave the best clinical efficacy. There were no significant differences in the stimulation parameters (output current amplitude, pulse width, stimulation frequency, VNS ON time and VNS OFF time) between the responder groups and non-responder groups (all p > 0.05, Table 2). In addition, there was no significant association between output current amplitude (r = −0.124, p = 0.335) and seizure reduction (%).

**Table 2:** Preoperative clinical characteristics and VNS settings at the end of one year follow up period in the responders_0_ and non-responders_0_ and the responders_50_ and non-responders_50_. AED, antiepileptic drug; CBZ, carbamazepine; OXCBZ, oxcarbazepine; GS, generalized seizure; FS, focal seizure; VNS, vagus nerve stimulation.

Variables	Responders_0_ (N = 55)	Non-responders_0_ (N = 8)	*P*_1_ value	Responders_50_ (N = 34)	Non-responders_50_ (N = 29)	*P*_2_ value
**Demographic data**						
Male/female	35/20	7/1	0.250	25/9	17/12	0.285
Age (years)	18.1 ± 8.6	20.3 ± 10.2	0.717	18.0 ± 9.1	18.8 ± 8.5	0.740
BMI (kg/m^2^)	21.9 ± 4.5	23.2 ± 3.4	0.364	21.8 ± 4.5	22.2 ± 4.4	0.836
**Daily activity**						
Sleeping overnight (hour)	8.3 ± 1.1	9.3 ± 1.2	0.119	7.9 ± 1.0	8.4 ± 1.3	0.131
Sitting quietly or lying quietly (hour)	3.5 ± 2.1	3.5 ± 1.6	0.893	3.5 ± 2.1	3.5 ± 2.6	0.614
Sitting busy (hour)	4.8 ± 1.9	5.2 ± 1.7	0.893	5.2 ± 1.9	4.9 ± 1.7	0.614
Light on-foot activities (hour)	5.2 ± 1.9	4.3 ± 1.8	0.498	5.2 ± 2.1	5.3 ± 1.7	0.956
Moderate on-foot activities (hour)	1.8 ± 1.0	1.7 ± 0.7	0.893	1.8 ± 1.0	1.8 ± 0.9	0.989
Strenuous activities (hour)	0.0 ± 0.0	0.0 ± 0.0	1.000	0.0 ± 0.0	0.0 ± 0.0	1.000
Very strenuous activities (hour)	0.0 ± 0.0	0.0 ± 0.0	1.000	0.0 ± 0.0	0.0 ± 0.0	1.000
**AED information**						
Number of AEDs	2.7 ± 1.2	2.0 ± 1.1	0.137	2.6 ± 1.4	2.6 ± 1.0	0.743
Daily dose (mg)	1702.6 ± 973.8	1501.7 ± 1276.2	0.337	1649.7 ± 1045.9	1709.3 ± 977.1	0.720
CBZ/OXCBZ/PHT	30(54.6%)	6(75.0%)	0.448	18(62.1%)	18(52.9%)	0.610
**Seizure characteristics**						
Epilepsy duration (years)	11.1 ± 7.4	10.8 ± 5.3	0.780	11.5 ± 7.3	10.6 ± 7.1	0.730
Seizures per month	98.5 ± 171.7	71.6 ± 138.9	0.901	125.6 ± 208.4	59.4 ± 90.6	0.730
FS	10(18.2%)	1(12.5%)	1.000	8(23.5%)	3(10.3%)	0.498
GS	21(38.2%)	1(12.5%)	0.243	14(41.2%)	8(27.6%)	0.498
GS + FS	24(43.6%)	6(75.0%)	0.136	12(35.3%)	18(62.1%)	0.225
**Etiology**						
Symptomatic	26(47.3%)	3(37.5%)	0.716	17(50.0%)	12(41.4%)	0.614
Cryptogenic	29(52.7%)	5(62.5%)	0.716	17(50.0%)	17(58.6%)	0.614
**VNS settings**						
Current amplitude (mA)	1.43 ± 0.53	1.26 ± 0.41	0.626	1.37 ± 0.57	1.48 ± 0.45	0.326
Pulse width (μs)	431.8 ± 112.4	389.0 ± 181.4	0.904	433.8 ± 112.0	431.0 ± 113.7	0.929
Frequency (Hz)	29.5 ± 1.6	23.9 ± 11.1	0.231	29.4 ± 1.6	28.8 ± 3.9	0.784
VNS ON time (s)	29.8 ± 1.2	26.7 ± 9.8	0.739	30.0 ± 0.0	29.7 ± 1.7	0.293
VNS OFF time (min)	5.4 ± 2.7	4.5 ± 1.4	0.739	5.0 ± 0.0	5.7 ± 3.7	0.293

### Holter data

**Figure 3:**
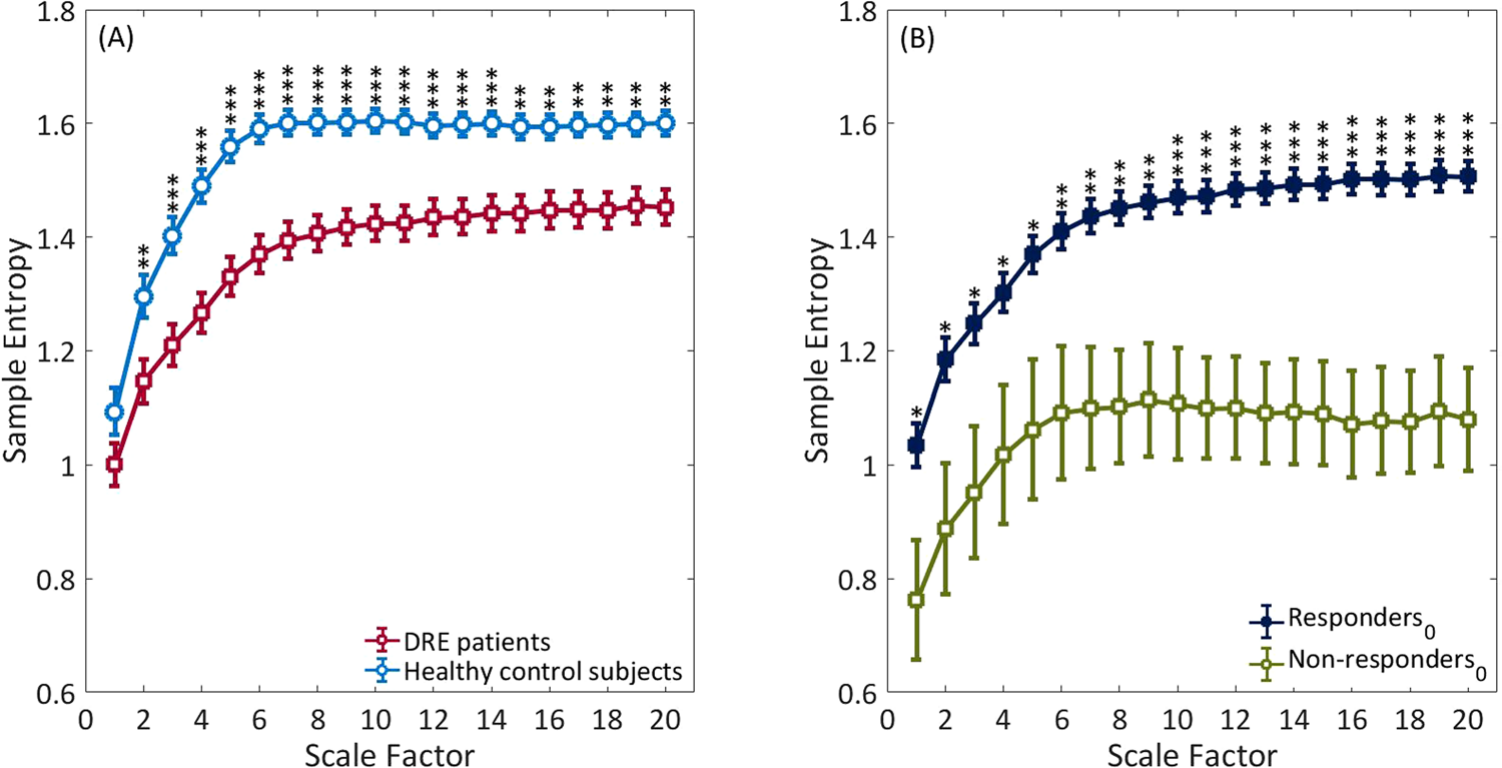
The sample entropy over different time scales. (**A**) The red square open symbols represented the entropy of patients with DRE before VNS, and the light blue open circles the entropy of healthy control subjects. (**B**) The dark blue solid squares represented the entropy of Responders_0_ before VNS, and the green open squares the entropy of Non-responders_0_ before VNS treatment. Symbols represent the mean values of entropy for each group and bars represent the standard error (${\rm{SE}}=\mathrm{SD}/\sqrt{n}$, where n is the number of subjects). *p < 0.05, **p < 0.01, and ***p < 0.001 for comparison between the responders_0_ and non-responders_0_.

The results of traditional HRV and MSE analyses in both groups are presented in Fig. 3 and Table 3. In awake state, the DRE patients exhibited significantly reduced entropy values over all time scales, except scale 1, in comparison with the healthy control group (Fig. 3). For traditional linear HRV parameters and CI derived from the MSE profiles, DRE patients had significantly lower Mean RR, SDNN, RMSSD, pNN50, VLF, LF, HF, TP, Slope 5, Area 1–5, Area 6–15 and Area 6–20 in comparison to the healthy control subjects (all p < 0.05, Table 3).

**Table 3:** Traditional HRV measurements and CI in patients with DRE and healthy control subjects.

Variables	DRE patients (n = 63)	Healthy control subjects (n = 50)	*P* value
**Traditional HRV analysis**			
Mean RR (msec)	713 ± 95	762 ± 85	0.003
SDNN (msec)	122 ± 36	157 ± 36	<0.001
RMSSD (msec)	34 ± 17	46 ± 19	<0.001
pNN50 (%)	11.58 ± 9.74	18.20 ± 10.13	<0.001
VLF (msec.^2^)	127 ± 84	208 ± 87	<0.001
LF (msec.^2^)	584 ± 422	1144 ± 750	<0.001
HF (msec.^2^)	618 ± 808	1073 ± 944	<0.001
TP (msec.^2^)	1331 ± 1175	2428 ± 1554	<0.001
LF/HF	1.42 ± 0.94	1.58 ± 1.04	0.397
**MSE analysis**			
Slope 5	0.08 ± 0.05	0.11 ± 0.06	0.001
Area 1–5	4.79 ± 1.12	5.52 ± 0.90	<0.001
Area 6–15	12.79 ± 2.23	14.40 ± 1.33	<0.001
Area 6–20	20.03 ± 3.45	22.39 ± 2.02	<0.001

For all the analyzed linear HRV parameters (Mean RR, SDNN, RMSSD, pNN50, VLF, LF, HF TP and LF/HF), there were no significant differences between the responders_0_ and non-responders_0_ group (all p > 0.05, Table 4). For non-linear parameters, the MSE profile were significantly higher in the responders_0_ group (Fig. 3). Meanwhile, the responders_0_ group had significantly higher Area 1–5 (4.94 ± 1.03 *vs*. 3.77 ± 1.30, p = 0.014), higher Area 6–15 (13.21 ± 1.88 *vs*. 9.90 ± 2.41, p = 0.001) and higher Area 6–20 (20.72 ± 2.86 *vs*. 15.30 ± 3.65, p < 0.001) of the CI derived from MSE profiles than non-responders_0_ group. The RMSSD (41 ± 20 *vs*. 27 ± 11, p = 0.001), pNN50 (15.54 ± 10.23 *vs*. 6.94 ± 6.73, p = 0.001), VLF (147 ± 92 *vs*. 107 ± 68, p = 0.028), LF (686 ± 463 *vs*. 465 ± 340, p = 0.026), HF (843 ± 1010 *vs*. 355 ± 330, p = 0.001) and TP (1678 ± 1403 *vs*. 924 ± 650, p = 0.005) were significantly higher and LF/HF (1.21 ± 0.87 *vs*. 1.66 ± 0.97, p = 0.040) were significantly lower in the patients who ultimately experienced significant seizure reductions ≥50%. In addition, there were no significant differences in CI between the responders_50_ and non-responders_50_ group (all p > 0.05, Table 4).

**Table 4:** Preoperative traditional HRV measurements and CI in the responders_0_ *vs*. non-responders_0_ and responders_50_ *vs*. non-responders_50_.

Variables	Responders_0_ (N = 55)	Non-responders_0_ (N = 8)	*P*_1_ value	Responders_50_ (N = 34)	Non-responders_50_ (N = 29)	*P*_2_ value
**Traditional HRV analysis**						
Mean RR (msec)	708 ± 93	748 ± 110	0.243	728 ± 94	696 ± 96	0.188
SDNN (msec)	120 ± 36	131 ± 43	0.628	127 ± 33	115 ± 39	0.136
RMSSD (msec)	35 ± 18	29 ± 9	0.327	41 ± 20	27 ± 11	0.001
pNN50 (%)	12.15 ± 10.01	7.65 ± 6.93	0.288	15.54 ± 10.23	6.94 ± 6.73	0.001
VLF (msec^2^)	128 ± 88	118 ± 55	0.812	147 ± 92	107 ± 68	0.028
LF (msec^2^)	597 ± 440	495 ± 272	0.628	686 ± 463	465 ± 340	0.026
HF (msec^2^)	659 ± 852	340 ± 268	0.151	843 ± 1010	355 ± 330	0.001
TP (msec^2^)	1386 ± 1234	954 ± 557	0.369	1678 ± 1403	924 ± 650	0.005
LF/HF	1.35 ± 0.87	1.93 ± 1.29	0.269	1.21 ± 0.87	1.66 ± 0.97	0.040
**MSE analysis**						
Slope 5	0.08 ± 0.05	0.08 ± 0.04	0.369	0.07 ± 0.05	0.09 ± 0.05	0.285
Area 1–5	4.94 ± 1.03	3.77 ± 1.30	0.014	5.03 ± 1.14	4.50 ± 1.05	0.099
Area 6–15	13.21 ± 1.88	9.90 ± 2.41	0.001	13.27 ± 2.05	12.21 ± 2.32	0.099
Area 6–20	20.72 ± 2.86	15.30 ± 3.65	<0.001	20.82 ± 3.09	19.11 ± 3.68	0.102

**Figure 4:**
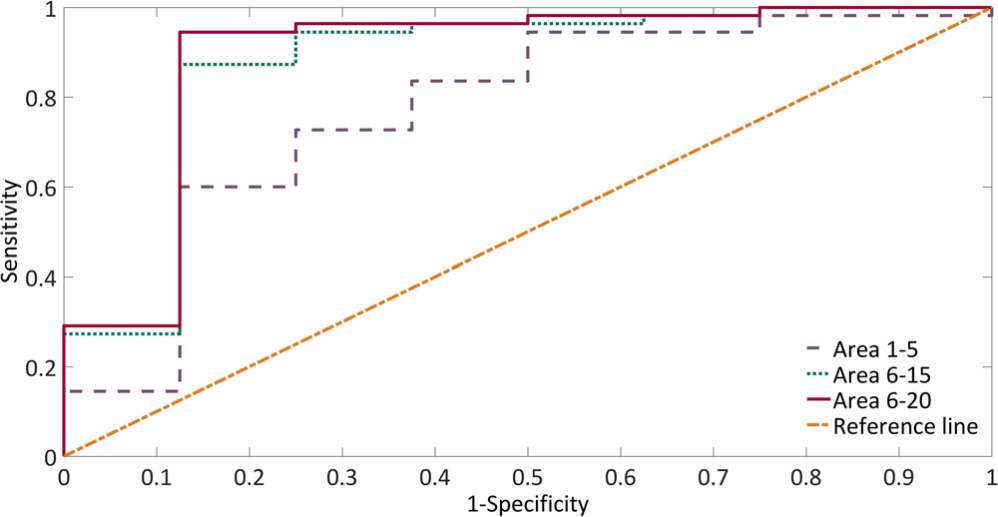
Analysis of the discrimination power of the responders_0_ and non-responders_0_ by receiver operating characteristic (ROC) curve analysis. The areas under the curve of Area 1–5, Area 6–15 and Area 6–20 were 0.770, 0.875 and 0.891, respectively.

**Figure 5:**
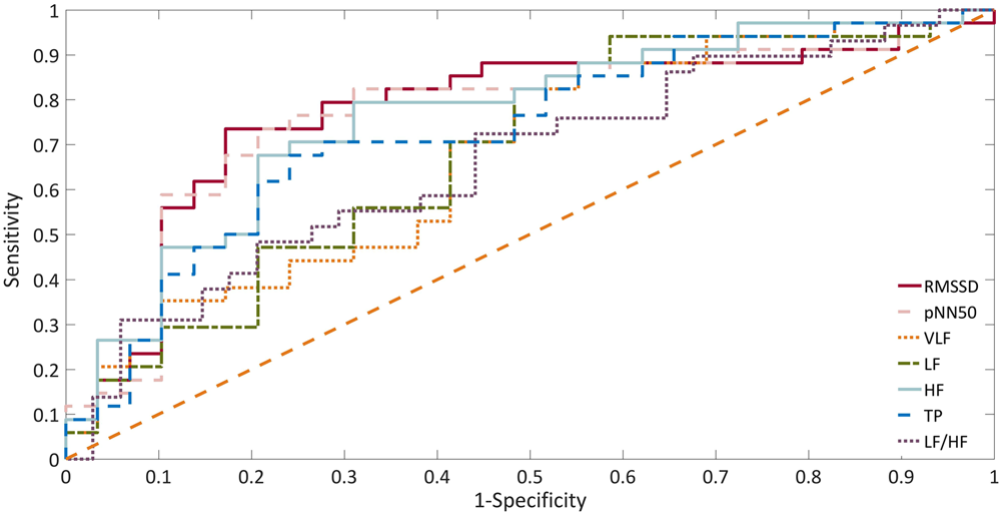
Analysis of the discrimination power of the responders_50_ and non-responders_50_ by receiver operating characteristic (ROC) curve analysis. The areas under the curve of RMSSD, pNN50, VLF, LF, HF, TP and LF/HF were 0.774, 0.766, 0.675, 0.682, 0.762, 0.729 and 0.662, respectively.

The ROC curves of the predictive parameters (Area 1–5, Area 6–15, Area 6–20, RMSSD, pNN50, VLF, LF, HF, TP and LF/HF) were depicted in Figs 4 and 5. Area 6–20 (AUC: 0.891 ± 0.082) showed the best overall discriminative power than Area 1–5 (AUC: 0.770 ± 0.100) and Area 6–15 (AUC: 0.875 ± 0.084) in the responders_0_ VNS outcome prediction. The sensitivity was 94.5% and the specificity was 87.5% when the Area 6–20 cut-off value was set at 16.82 (Fig. 4). The RMSSD (AUC: 0.774 ± 0.063) showed the best overall discriminative power with pNN50 (AUC: 0.766 ± 0.063), VLF (AUC: 0.675 ± 0.068), LF (AUC: 0.682 ± 0.069), HF (AUC: 0.762 ± 0.061), TP (AUC: 0.729 ± 0.064), and LF/HF (AUC: 0.662 ± 0.069) being slightly lower for responders_50_ seizure reduction prediction. Furthermore, the sensitivity was 79.4% and the specificity was 72.4% when the RMSSD cut-off was set at 30 msec (Fig. 5).

In addition, the CI including Area 1–5 (r = 0.258, p = 0.041), Area 6–15 (r = 0.304, p = 0.015), and Area 6–20 (r = 0.320, p = 0.011) as well as traditional linear HRV measurements containing RMSSD (r = 0.334, p = 0.007), pNN50 (r = 0.289, p = 0.022), VLF (r = 0.267, p = 0.035), LF (r = 0.254, p = 0.045) and TP (r = 0.272, p = 0.031) significantly positively correlated with seizure reduction (%). In multivariable logistic regression analysis, Area 6–20 (β = 0.320, p = 0.011) and RMSSD (β = 0.334, p = 0.007) were independent factors associated with seizure reduction (%) in the final responders_0_ and responders_50_ model, respectively.

## Discussion

Although VNS has been extensively used as an effective therapy for DRE, it is still not possible to predict which patients will respond to VNS treatment and to what extent. To the best of our knowledge, this is the first study focusing on predicting seizure reduction of VNS treatment based on preoperative ECG characteristic indices and heart rhythm complexity using traditional linear HRV analyses and MSE method in patients with DRE. We found that a lower heart rhythm complexity, quantified by MSE, is associated with the unresponsiveness (non-responders_0_) to VNS treatment. Furthermore, VNS responders (responders_50_) tend to show relatively less severe cardiac autonomic dysfunction. In the correlation and regression study, the seizure reduction (%) correlated with the CI of heart rhythm complexity and HRV measurements, but not output current of vagus nerve stimulator. In addition, the ROC analysis revealed that preoperative Area 6–20 and RMSSD had the greatest discriminatory power to detect the responders_0_ and responders_50_ with high sensitivity and specificity when specific cut-off values are applied.

In the present study, CI and all of the analyzed traditional HRV measures were significantly lower in the DRE patients than in those healthy control subjects except for the LF/HF. The results confirm that DRE patients have dysregulated cardiac autonomic function as well as impaired heart rhythm complexity. Most studies showed that seizure characteristics were prognostic predictors of VNS therapy in patients with DRE^25–30^. Englot *et al*. found that generalized epilepsies received more benefit than those with partial seizures, though complete seizure control was rarely obtained^27,28,30^. In contrast, Labar showed that Lennox-Gastaut syndrome was more likely to show unresponsiveness to VNS therapy^31^. However, another study by Englot *et al*. demonstrated that patients with predominantly partial seizures responded most favorably to VNS, whereas those with generalized tonic-clonic seizures responded least favorably. They also concluded that a longer duration of epilepsy was somewhat predictive of poorer response to VNS^28^. As with Englot’s result, Helmers and Ranfroe also concluded that longer duration epilepsy patients had less possibility of complete seizure control through VNS treatment^32,33^. On the contrary, Labar concluded that a longer epilepsy duration was an independent indicator of VNS responsiveness^31^. Similarly, analyses of the relationship between the baseline seizure frequency and the VNS responsiveness have shown contradictory results^34–36^. Several studies also attempted to predict the success of VNS based on electroencephalography (EEG) and magnetic resonance imaging (MRI) data. According to a recent study, presurgical EEG symmetry quantified by pair wise derived brain symmetry index (pdBSI) showed promising results in predicting responsiveness to VNS treatment^37^. One study focusing on the use of EEG before VNS showed that patients with no bilateral interictal epileptiform discharges (IED) had a significantly higher chance of being seizure-free on VNS therapy than those with IED^26^. However, Arcos *et al*. had contradictory results that epilepsy patients with a temporal region discharge were more likely to respond^25^. Furthermore, Janszky *et al*. found that malformation of the cortical development as seen in an MRI was associated with successful VNS treatment according to a single predictive variable analysis^26^. Arcos *et al*. also concluded that seizure outcomes were positively related to lesions indicated by MRI measurements^25^. The present study showed that there were no significant differences in demographic data, AED regimens, seizure characteristics, etiology and VNS settings for the responders_0_ and non-responders_0_ group. Though many predictors and potential factors of response to VNS treatment in patients with DRE have been proposed, their feasibility requires support for clinical data, and predictors of success are still elusive.

Traditional time domain and frequency domain analyses of HRV is a useful tool to evaluate the cardiac autonomic function, and therefore is commonly used in health risk stratification and efficacy prediction^22–24^. Since DRE is characterized by recurrent and unprovoked seizures that seem to be associated with cardiac autonomic dysfunction^8,9^, preoperative HRV characteristics could be potential biomarkers to predict long-term treatment outcome. Persson *et al*. reported that temporal lobe epileptic patients with a poor craniotomy surgery outcomes had more pronounced impairment of sympathetic as well as parasympathetic cardiac control than those with good outcomes^38^. Subsequently, the potential relationship between craniotomy surgery outcomes and preoperative HRV parameters was revealed for the first time. In our previous study, we showed that DRE patients with higher parasympathetic cardiac control or vagal tone are more likely to respond to VNS treatment^39^. The present study shows consistently results that the VNS treatment response (responder_50_) is significantly associated with the degree of preoperative vagal activity represented by RMSSD, pNN50 and HF, with more positive effects in patients with higher vagal cardiac control. However, traditional linear HRV analyses to predict VNS outcome, may not reflect the true cardiac autonomic regulation and complexity of the heart rate dynamics. Furthermore, no studies have evaluated whether the responsiveness to VNS can be predicted using a baseline complexity of HRV.

Complexity is a concept that lies between periodicity and randomness, and the decrease of complexity under free-running conditions reflects a declined ability of the systems to function in certain dynamical regimes, possibly due to dysregulation or impairment of control mechanisms^26^. Previous studies that focused on MSE analysis of EEG signals in patients with epilepsy found dynamical changes of EEG complexity, confirming that the analysis of MSE can provide a quantifiable and accurate method to investigate patients with seizures, providing a promising biomarker^40–42^. Furthermore, the prognostic value of heart rhythm complexity quantified by MSE were studied and confirmed in patients with congestive heart failure, acute stroke and permanent atrial fibrillation^13,14,43^. The present study clearly demonstrated that the values of traditional linear HRV parameters calculated by time and frequency domain analyses were comparable in the responders_0_ and non-responders_0_ groups. Since the fluctuations in the time intervals between adjacent heartbeats is an emergent property of interdependent regulatory systems operating non-linearly on different time scales, traditional linear HRV analyses may be inadequate to characterize and reveal the underlying multiscale interacting mechanisms of interbeat interval dynamics. Though traditional linear HRV indices are often used to assess the cardiac autonomic function^22^, data from the present study did not provide further evidence to support responders_0_ having relative better cardiac autonomic function than the non-responders_0_. Nevertheless, the MSE method has shown to be a novel analytical tool for predicting success of VNS treatment. Consistent with our findings, Ho *et al*. also showed that heart rhythm complexity has a better prognostic power in patients with congestive heart failure^43^. Furthermore, our results showed that the responders_0_ group had significantly higher CI including Area 1–5, Area 6–15 and Area 6–20 before VNS surgery. The significant positive association between seizure reduction and Area 6–20 implied a direct association between VNS outcomes and baseline heart rhythm complexity.

According to previous studies, the CI including Slope 5 and Area 1–5 at small scales in MSE probes the complexity structure of the heart rate dynamics and may give a powerful overall estimation of heart rate short-term complexity and the integrity of sinus arrhythmia^11,16,17^, while CI (Area 6–15 and Area 6–20) of large time scales are more controversial since several physiological mechanisms such as sympathetic, baroreflex and hormonal regulation beneath these time scales^16,17^. In the present study, results of ROC curve analysis showed that preoperative Area 6–20 had the greatest discriminatory power to differentiate the non-responders_0_ from the responders_0_. Two previous studies also identified Area 6–20 as independent risk stratification for the prognosis of patients with acute ischemic stroke and congestive heart failure, respectively^13,43^. The Area 6–20 derived from MSE were significantly lower in the non-responder_0_ group in our study, and this phenomenon about lower Area 6–20 indicating bad prognosis was consistent with those found by Ho *et al*. and Chen *et al*. in MSE study^13,43^. Dynamical fluctuations of the HRV signal originated from the cardiovascular system with multiple interacting components usually exhibit remarkably complicated patterns over different time scales^11,16^. Although the underlying control mechanisms were still not clear, it is possible that the heart rhythm complexity at large time scales (from scale 6 to 20) is originated by the heart itself. This intrinsic feature of heart that seems to be essential for keep healthy and more accurately reflects the underlying heart rate dynamics. The present preliminary study provided a unique window into the VNS treatment prognosis of DRE by exploring the dynamical complexity on the system level. Our results imply that the MSE analysis might be applicable in predicting success of VNS treatment for patients with DRE.

Several limitations are presented within this study. Firstly, though we stabilized the AED regimen of DRE patients during the one year follow up period, it is still difficult to discriminate between the effects of the AED and the VNS. In addition, the potential effects of different AEDs on the period of heartbeat time series selected for HRV analysis were not completely excluded. Secondly, we recruited heterogeneous DRE with a wide age range, difference in type of seizures and various localization/lateralization of epileptic focus, the potential effects of focus lateralization, seizure type and age on VNS outcome prediction should be elucidated in future studies. Thirdly, although the ECG recordings were carefully acquired and analyzed to reduce errors introduced by experimental method, the heterogeneity of the patients, their mental workloads, and the recording environments may contribute to differences in the MSE and CI between the responders_0_ and non-responders_0_. Recording all the ECG data in free running conditions may also introduce variations with possible confounding factors such as stress, emotion, and breathing patterns. These findings are preliminary because they are based on a study with a non-controlled small sample size of DRE patients. To establish the relationship between VNS outcome and heart rhythm complexity will require a multicenter, sizeable and prospective study.

## Conclusions

In conclusion, this multicenter preliminary study suggests that preoperative heart rhythm complexity is useful to predict the unresponsiveness (non-responders_0_) to VNS treatment. Furthermore, indices of linear HRV preoperatively demonstrate that responders_50_ of VNS have a less impairment of parasympathetic cardiac control or vagal tone than those of the non-responers_50_. The potential physiological interpretations of these findings for the prognosis of VNS treatment remains to be elucidated, but this knowledge about CI and linear HRV measurements as non-invasive biomarkers of predicting seizure reduction of VNS is important for optimizing patient selection in a more objective way and counselling patients to avoid unnecessary VNS surgeries in non-responders_0_ or non-responders_50_ and to improve the overall clinical efficacy of VNS treatment. This investigation may also facilitate other studies recruiting larger DRE patients sample size to more clearly show the heart rate dynamics of appropriate VNS candidates.

## Electronic supplementary material

Table S-1
