# Supplementary material for: Preoperative Heart Rate Variability as Predictors of Vagus Nerve Stimulation Outcome in Patients with Drug-resistant Epilepsy
Source: Sci Rep. 2018 Mar 1;8:3856. doi: 10.1038/s41598-018-21669-3 (PMC5832772; doi:10.1038/s41598-018-21669-3)
Supplement: Supplementary file 1 — Table S-1 [file 41598_2018_21669_MOESM1_ESM.pdf]

Preoperative Heart Rate Variability as Predictors of Vagus Nerve Stimulation Outcome in Patients with Drug-resistant Epilepsy

Hong-Yun Liu[1, 2], Zhao Yang[1], Fan-Gang Meng[3, 4], Yu-Guang Guan[5], Yan-Shan Ma[6], Shu-Li Liang[7], Jiu-Luan Lin[8], Long-Sheng Pan[7], Ming-Ming Zhao[9], Wei Qu[1], Hong-Wei Hao[1], Guo-Ming Luan[5], Jian-Guo Zhang[4], Luming Li[1, 10, 11, 12*]

[1]National Engineering Laboratory for Neuromodulation, School of Aerospace Engineering, Tsinghua University, 100084 Beijing, China

[2]Department of Biomedical Engineering, Chinese PLA General Hospital, Fuxing Road, 100853 Beijing, China

[3]Beijing Neurosurgical Institute, 100050, Beijing, China

[4]Neurosurgery, Beijing Tian Tan Hospital Capital Medical University, 100050, Beijing, China

[5]Neurosurgery, Sanbo Brain Hospital Capital Medical University, 100093, Beijing, China

[6]Neurosurgery, Peking University First Hospital FengTai Hospital, 100071, Beijing, China

[7]Neurosurgery, Chinese PLA General Hospital, Fuxing Road, 100853 Beijing, China

[8]Neurosurgery, TsingHua University YuQuan Hospital, 100040, Beijing, China

[9]Neurosurgery, Navy General Hospital, 100048, Beijing, China

[10]Man-Machine-Environment Engineering Institute, School of Aerospace Engineering, Tsinghua University, 100084 Beijing, China

[11]Precision Medicine & Healthcare Research Center, Tsinghua-Berkeley Shenzhen Institute, 518055, Shenzhen, China

[12]Center of Epilepsy, Beijing Institute for Brain Disorders, 100069, Beijing, China

*lilm@mail.tsinghua.edu.cn

Patient Number	Gender	Age (year)	Number of AEDs	AEDs and daily dose (mg)	Ictal scalp EEG characteristics	Seizure types	Etiology	Epilepsy duration (year)	VNS settings	Outcome	Seizure reduction
P1	M	19	3	CBZ(900),VPA(1000),PHT(450)	Bilateral temporal abnormalities	GS, FS	Cryptogenic	14	1.6mA,500μs,30Hz,30s ON, 5min OFF	Non-responder	27.78%
P2	M	11	3	VPA(1000),CBZ(600),TPM(50)	Left parietal,occipital,temporal focus	FS	Symptomatic (left occipital lobe atrophy)	5	2.0mA,500μs,30Hz,30s ON, 5min OFF	Responder	52.83%
P3	M	7	4	TPM(12.5),VPA(500),CZP(1.25),LTG(100)	Bilateral frontal,temporal focus	GS, FS	Cryptogenic	5	1.7mA,500μs,30Hz,30s ON, 5min OFF	Non-responder	0.00%
P4	M	19	1	LEV(1000)	Left temporal focus	FS	Cryptogenic	11	1.4mA,500μs,30Hz,30s ON, 5min OFF	Responder	100.00%
P5	F	27	4	LEV(1500),PHB(90),LTG(200),VPA(1000)	Bilateral frontal,temporal focus	GS, FS	Symptomatic (postencephalitis)	2	2.0mA,500μs,30Hz,30s ON, 5min OFF	Non-responder	25.00%
P6	M	22	2	OXCBZ(900),LEV(1000)	Bilateral frontal,temporal focus	GS, FS	Symptomatic(white matter myelination)	12	2.4mA,500μs,30Hz,30s ON, 5min OFF	Responder	91.67%
P7	M	33	1	CBZ(600)	Bilateral temporal abnormalities	GS, FS	Symptomatic (right hippocampal atrophy)	17	1.4mA,500μs,30Hz,30s ON, 5min OFF	Non-responder	0.00%
P8	M	21	6	MGVPA(500),DZP(20),PHT(300),OXCBZ(900),LTG(150),TCM(2800)	Bilateral frontal,temporal focus	GS	Cryptogenic	20	1.5mA,500μs,30Hz,30s ON, 5min OFF	Responder	63.64%
P9	F	24	3	LTG(200),LEV(2000),OXCBZ(1500)	Bilateral temporal abnormalities	GS	Symptomatic (encephalomalicia)	12	1.6mA,500μs,30Hz,30s ON, 5min OFF	Responder	96.67%
P10	F	28	2	VPA(1000),LTG(150)	Bilateral temporal,occipital focus	GS	Cryptogenic	11	2.6mA,500μs,30Hz,30s ON, 5min OFF	Non-responder	44.44%
P11	F	19	1	VPA(1000)	Left frontal,parietal;Right temporal, parietal focus	GS	Symptomatic (encephalomalicia & craniocerebral trauma)	5	0.8mA,500μs,30Hz,30s ON, 5min OFF	Responder	87.50%
P12	M	27	2	PHB(180),CBZ(1800)	Non-specific EEG abnormalities	GS	Cryptogenic	21	1.3mA,500μs,30Hz,30s ON, 5min OFF	Non-responder	4.44%
P13	F	25	1	VPA(400)	Left occipital,bilateral temporal abnormalities	GS, FS	Symptomatic (right hippocampal atrophy)	10	1.9mA,500μs,30Hz,30s ON, 5min OFF	Non-responder	30.77%
P14	M	18	2	LEV(250),VPA(1000)	Right temporal abnormalities	GS, FS	Symptomatic (left hippocampal sclerosis)	10	1.3mA,500μs,30Hz,30s ON, 5min OFF	Non-responder	37.50%
P15	F	19	4	CBZ(1100),LEV(2000),PHB(45),LTG(150)	Bilateral frontal, parietal, temporal abnormalities	GS, FS	Symptomatic (postencephalitis)	4	1.5mA,500μs,30Hz,30s ON, 5min OFF	Non-responder	42.86%
P16	M	7	1	CBZ(400)	Left parietal focus	FS	Symptomatic (tuberous sclerosis)	5	1.4mA,500μs,30Hz,30s ON, 5min OFF	Responder	100.00%
P17	M	32	1	VPA(1500)	Non-specific EEG abnormalities	GS	Cryptogenic	30	1.6mA,500μs,30Hz,30s ON, 5min OFF	Responder	100.00%
P18	M	21	1	VPA(1500)	Right temporal focus	GS, FS	Symptomatic (encephalomalicia)	11	1.0mA,500μs,30Hz,30s ON, 5min OFF	Responder	98.89%
P19	M	38	2	CBZ(600),MGVPA(500)	Right temporal, bilateral frontal abnormalities	GS, FS	Symptomatic(postoperative glioma)	16	1.4mA,500μs,30Hz,30s ON, 5min OFF	Non-responder	0.00%
P20	F	21	1	CBZ(800)	Right parietal, temporal focus	FS	Cryptogenic	9	1.2mA,500μs,30Hz,30s ON, 5min OFF	Responder	83.33%
P21	M	31	2	MGVPA(1000),CPNCM(1000)	Right frontal, temporal abnormalities	GS, FS	Symptomatic (encephalomalicia)	29	0.9mA,500μs,30Hz,30s ON, 5min OFF	Responder	98.03%
P22	M	12	2	OXCBZ(450),ZNS(300)	Non-specific EEG abnormalities	GS, FS	Symptomatic (heterotopias)	10	0.8mA,500μs,30Hz,30s ON, 5min OFF	Responder	100.00%
P23	M	25	2	CBZ(200),LTG(50)	Right frontal,parietal,temporal abnormalities	GS, FS	Symptomatic(pachygyria abnormality)	12	1.0mA,500μs,30Hz,30s ON, 5min OFF	Responder	100.00%
P24	M	10	3	LTG(62.5),LEV(1500),VPA(18)	Non-specific EEG abnormalities	FS	Cryptogenic	7	1.7mA,500μs,30Hz,30s ON, 5min OFF	Responder	71.43%
P25	F	7	3	LEV(750),VPA(500),TPM(150)	Bilateral temporal,Right parietal focus	GS	Symptomatic (encephalomalicia)	3	1.0mA,500μs,30Hz,30s ON, 5min OFF	Non-responder	33.33%
P26	F	11	2	LTG(250),OXCBZ(450)	Non-specific EEG abnormalities	GS, FS	Cryptogenic	10	1.3mA,500μs,30Hz,30s ON, 5min OFF	Non-responder	27.27%
P27	F	10	4	LTG(50),CPNCM(600),PMT(250),MGVPA(500)	Left temporal abnormalities	FS	Symptomatic (left hippocampal abnormalties)	8	1.7mA,500μs,30Hz,30s ON, 5min OFF	Responder	70.00%
P28	M	15	2	CBZ(800),VPA(2000)	Left parietal,occipital, Bilateral temporal focus	GS, FS	Symptomatic (toxicosis)	4	1.5mA,500μs,30Hz,30s ON, 5min OFF	Non-responder	0.00%
P29	M	6	3	VPA(750),LTG(100),TPM(150)	Left frontal,parietal,temporal focus	FS	Symptomatic (focal cortical dysplasia)	2	1.2mA,500μs,30Hz,30s ON, 5min OFF	Responder	82.50%
P30	M	34	1	CBZ(1400)	Bilateral temporal, frontal abnormalities	GS, FS	Symptomatic (encephalomalicia & atrophy)	34	2.2mA,500μs,30Hz,30s ON, 5min OFF	Non-responder	44.09%
P31	F	10	3	OXCBZ(375),ZNS(200),CZP(2)	Non-specific EEG abnormalities	FS	Cryptogenic	5	1.5mA,500μs,30Hz,30s ON, 5min OFF	Non-responder	20.00%
P32	M	7	4	OXCBZ(900),TPM(150),PHB(60),CZP(3)	Non-specific EEG abnormalities	GS, FS	Cryptogenic	6	2.4mA,500μs,30Hz,30s ON, 5min OFF	Responder	64.11%
P33	M	18	3	OXCBZ(600),TPM(100),VPA(1000)	Non-specific EEG abnormalities	GS, FS	Cryptogenic	14	1.8mA,250μs,30Hz,30s ON, 5min OFF	Non-responder	0.00%
P34	F	30	3	VPA(1000),LTG(200),CBZ(600)	Bilateral temporal abnormalities	GS, FS	Cryptogenic	16	1.7mA,500μs,25Hz,30s ON, 5min OFF	Responder	96.67%
P35	M	6	2	VPA(500),LEV(625)	Bilateral parietal, occipital abnormalities	GS	Symptomatic (focal cortical dysplasia)	6	1.8mA,500μs,25Hz,30s ON, 5min OFF	Responder	75.00%
P36	M	7	2	LTG(100),LEV(1250)	Bilateral parietal, occipital abnormalities	GS, FS	Symptomatic (focal cortical dysplasia)	6	1.4mA,500μs,25Hz,30s ON, 25min OFF	Non-responder	25.00%
P37	M	17	3	VPA(1500),CBZ(600),LTG(300)	Non-specific EEG abnormalities	GS	Symptomatic (abnormal lateral ventricles)	9	1.5mA,500μs,25Hz,30s ON, 5min OFF	Responder	60.00%
P38	M	8	3	LEV(750),VPA(560)，LTG(125)	Right temporal,parietal focus	GS, FS	Cryptogenic	5	2.0mA,250μs,25Hz,21s ON, 5min OFF	Non-responder	40.00%
P39	F	12	2	CBZ(200),VPA(1000)	Bilateral frontal, temporal abnormalities	GS	Cryptogenic	10	2.1mA,500μs,25Hz,30s ON, 5min OFF	Responder	96.67%
P40	M	18	1	OXCBZ(450)	Left parietal,temporal, occipital abnormalities	GS	Cryptogenic	15	0.7mA,500μs,25Hz,30s ON, 5min OFF	Non-responder	0.00%
P41	M	30	3	TPM(100),OXCBZ(1200),PHB(90)	Left frontal, temporal, parietal focus	GS	Cryptogenic	29	1.4mA,500μs,30Hz,30s ON, 5min OFF	Responder	100.00%
P42	M	22	4	LTG(2500),TPM(200),VPA(1500), CZP(4)	Non-specific EEG abnormalities	FS	Cryptogenic	16	1.0mA,500μs,30Hz,30s ON, 5min OFF	Responder	99.50%
P43	F	25	2	LTG(2750),VPA(1250)	Non-specific EEG abnormalities	GS, FS	Cryptogenic	25	0.9mA,500μs,30Hz,30s ON, 5min OFF	Responder	75.00%
P44	M	21	4	VPA(1250),CBZ(800),CZP(2)，LTG(75)	Right frontal, temporal abnormalities	GS	Cryptogenic	19	1.5mA,500μs,30Hz,30s ON, 5min OFF	Non-responder	27.42%
P45	M	10	2	TPM(200),VPA(1000)	Right occipital focus	GS	Cryptogenic	10	1.8mA,500μs,30Hz,30s ON, 5min OFF	Responder	50.00%
P46	M	17	3	LTG(100),LEV(1500),VPA(1500)	Non-specific EEG abnormalities	GS	Cryptogenic	4	1.5mA,250μs,30Hz,30s ON, 5min OFF	Non-responder	33.33%
P47	M	30	1	CBZ(600)	Bilateral temporal, Right frontal abnormalities	GS	Cryptogenic	11	0.5mA,250μs,30Hz,30s ON, 5min OFF	Responder	100.00%
P48	F	14	1	OXCBZ(750)	Left frontal focus	FS	Cryptogenic	8	1.2mA,500μs,10Hz,30s ON, 5min OFF	Non-responder	0.00%
P49	M	19	3	CBZ(600),TPM(250),VPA(1250)	Non-specific EEG abnormalities	GS, FS	Cryptogenic	13	2.5mA,250μs,30Hz,30s ON, 5min OFF	Responder	60.00%
P50	M	14	3	VPA(1500),OXCBZ(900),PHB(45)	Bilateral temporal abnormalities	GS	Cryptogenic	13	1.0mA,250μs,30Hz,30s ON, 5min OFF	Non-responder	16.67%
P51	M	19	2	VPA(1500),PHT(200)	Bilateral frontal, parietal, temporal abnormalities	GS, FS	Cryptogenic	7	1.0mA,250μs,30Hz,30s ON, 5min OFF	Responder	57.14%
P52	M	8	6	VPA(600),OXCBZ(450),LTG(100),CZP(2),PHT(150),PHB(60)	Non-specific EEG abnormalities	GS, FS	Cryptogenic	5	1.0mA,250μs,30Hz,30s ON, 5min OFF	Responder	50.00%
P53	F	5	1	TPM(150)	Bilateral temporal abnormalities	GS	Symptomatic (abnormal lateral ventricles)	5	1.0mA,250μs,30Hz,30s ON, 5min OFF	Responder	91.11%
P54	M	26	4	CBZ(200),DZP(5),PHT(200),TCM(200)	Left temporal, Right parietal, Bilateral occipital abnormalities	GS, FS	Symptomatic (right cerebellar atrophy)	18	0.4mA,250μs,30Hz,30s ON, 5min OFF	Non-responder	33.00%
P55	F	11	3	OXCBZ(1500),TPM(225),CZP(1)	Bilateral parietal, occipital, temporal abnormalities	GS	Symptomatic (focal cortical dysplasia)	2	1.8mA,250μs,30Hz,30s ON, 5min OFF	Non-responder	37.50%
P56	M	11	3	VPA(750),LTG(1500),LEV(1000)	Bilateral occipital focus	GS	Symptomatic (focal cortical dysplasia)	5	2.0mA,250μs,30Hz,30s ON, 5min OFF	Responder	96.67%
P57	M	26	3	VPA(500),GBP(200),PHB(15)	Bilateral frontal focus	GS	Cryptogenic	11	0.2mA,250μs,30Hz,30s ON, 5min OFF	Responder	100.00%

P58	M	37	2	MGVPA(1000),CPNCM(800)	Bilateral temporal, Right frontal abnormalities	GS, FS	Symptomatic (encephalomalicia)	14	0.5mA,250μs,30Hz,30s ON, 5min OFF	Responder	70.00%
P59	F	12	3	VPA(750),OXCBZ(975),LTG(1000)	Non-specific EEG abnormalities	FS	Cryptogenic	7	1.8mA,500μs,30Hz,30s ON, 5min OFF	Non-responder	33.33%
P60	F	6	5	LTG(1000),CBZ(150),VPA(600),PHB(30),CZP(0.75)	Bilateral parietal, occipital abnormalities	GS	Symptomatic (focal cortical dysplasia)	6	1.0mA,250μs,30Hz,30s ON, 5min OFF	Responder	100.00%
P61	F	27	3	TPM(300),CBZ(800),VPA(1500)	Left temporal, parietal focus	GS, FS	Cryptogenic	10	1.2mA,500μs,30Hz,30s ON, 5min OFF	Non-responder	33.33%
P62	F	15	3	LTG(1000),VPA(1000),TPM(150)	Bilateral parietal, occipital abnormalities	GS, FS	Cryptogenic	13	1.5mA,250μs,30Hz,30s ON, 5min OFF	Non-responder	25.00%
P63	M	19	2	VPA(2000),LTG(2000)	Non-specific EEG abnormalities	GS, FS	Cryptogenic	7	1.0mA,250μs,30Hz,30s ON, 5min OFF	Non-responder	0.00%

Table S-1. Clinical and therapeutic data of 63 patients with drug-resistant epilepsy. AED, antiepileptic drug; VPA, valproate; LTG, lamotrigine; CBZ, carbamazepine; OXCBZ, oxcarbazepine; LEV, levetiracetam; TPM, topiramate; PHB, phenobarbital; CZP, clonazepam; PHT, phenytoin; MGVPA, magnesium valproate; CPNCM, compound phenobarbital nitrazepam and chlorphenamine maleate; DZP, diazepam; ZNS, zonisamide; TCM: traditional Chinese medicine; PMT, primidone; GBP, gabapentin; VNM, valnromide; GS, generalized seizure; FS, focal seizure.
